# Dopamine systems adaptation during acquisition and consolidation of a skill

**DOI:** 10.3389/fnint.2014.00087

**Published:** 2014-11-05

**Authors:** Wolfgang H. Sommer, Rui M. Costa, Anita C. Hansson

**Affiliations:** ^1^Institute of Psychopharmacology at Central Institute of Mental Health, University of HeidelbergMannheim, Germany; ^2^Department of Addiction Medicine at Central Institute of Mental Health, University of HeidelbergMannheim, Germany; ^3^Champalimaud Neuroscience Programme, Champalimaud Center for the UnknownLisbon, Portugal

**Keywords:** dopamine receptors, receptor binding, rotarod, striatum, gene expression, neuroadaptation, learning

## Abstract

The striatum plays a key role in motor learning. Striatal function depends strongly on dopaminergic neurotransmission, but little is known about neuroadaptions of the dopamine system during striatal learning. Using an established task that allows differentiation between acquisition and consolidation of motor learning, we here investigate D1 and D2-like receptor binding and transcriptional levels after initial and long-term training of mice. We found profound reduction in D1 binding within the dorsomedial striatum (DMS) after the first training session on the accelerated rotarod and a progressive reduction in D2-like binding within the dorsolateral striatum (DLS) after extended training. Given that similar phase- and region-specific striatal neuroadaptations have been found also during learning of complex procedural tasks including habit formation and automatic responding, the here observed neurochemical alterations are important for our understanding of neuropsychiatric disorders that show a dysbalance in the function of striatal circuits, such as in addictive behaviors.

## Introduction

The ability to optimize learned motor sequences is essential for survival. In fact, most of our behaviors are organized in orderly structured actions consolidated into reflex-like response patterns that are largely resistant to interference (Shiffrin and Schneider, 1984; Dickinson, 1985). Mostly, we are unaware of these actions. If the behavioral patterns are more complex and come occasionally to our attention, we think of them as habits. Running automatically through an action sequence frees our mind for other tasks that require attention. However, such perfectionism has an important down side: it is difficult to overcome. After a smoker has gone through the same sequence of events many thousand times, he or she is often not aware of lightening a cigarette. A distinct cue may have triggered the event independent from any desire. Maladaptive automated processes are seen as key components in the development of pathological behaviors including addictive disorders (Everitt and Robbins, 2005).

Two key stages can be identified in the formation of a well-established action sequence, an initial acquisition phase, characterized by a steep learning curve, and a phase of more gradual improvements, in which the behavior is optimized and becomes less susceptible to external influences (Shiffrin and Schneider, 1984; Karni et al., 1998; Muellbacher et al., 2002; Kleim et al., 2004). These two phases of motor learning can be distinguished in a simple rodent model, the accelerating rotarod task (Costa et al., 2004; Luft and Buitrago, 2005; Yin et al., 2009). Previous studies have found phase-specific changes in neural activity in the dorsal striatum and other regions of the basal ganglia circuitry (Jenkins et al., 1994; Carelli et al., 1997; Ungerleider et al., 2002; Costa et al., 2004; Barnes et al., 2005).

A defining feature of the dorsal striatum (caudate putamen, CPu) is the existence of two, roughly equally sized populations of medium spiny GABAergic neurons expressing either dopamine (DA) D1 or D2 receptors (Gerfen et al., 1990). These two populations account for nearly 90% of all neurons in this region. They project either directly (D1 neurons) or indirectly (D2 neurons) to the substantia nigra pars reticulata (SNR), a midbrain structure that relays information from the basal ganglia motor circuitry to the thalamus. The SNR resides in close proximity to the origin of dopaminergic input to the dorsal striatum, i.e., the dopaminergic neurons of the substantia nigra pars compacta (SNC). The basal ganglia motor circuitry is essential in controlling voluntary movements whereby the direct D1 pathway is typically seen as the driver, while the indirect D2 pathway was ascribed the role of the break, together ensuring the fine tuning of intentional motor activity (Albin et al., 1989; Alexander and Crutcher, 1990; Sommer et al., 1996; Gerfen and Surmeier, 2011). Although this dichotomous model is still under discussion (Cui et al., 2013), coordinated activity in both pathways is essential for proper action selection and execution (Chan et al., 2005; Brown, 2007; Gremel and Costa, 2013).

The dorsal striatum receives inputs from most cortices (McGeorge and Faull, 1989; Voorn et al., 2004; Pan et al., 2010). An anatomical and functional mediolateral distinction exists in the dorsal striatum inasmuch as regions receiving input primarily from association cortices (i.e., associative or dorsomedial striatum, DMS), are more involved during the initial learning phase, while the parts of the striatum connected to the sensorimotor cortex (i.e., sensorimotor or dorsolateral striatum, DLS) are predominantly engaged in later consolidation and habit formation (Yin et al., 2004, 2005, 2009).

While some insights have been gained concerning glutamatergic plasticity during the early and late phase of motor learning on the accelerated rotarod (Yin et al., 2009), neuroadaptations within the DA system have not been studied so far. Here, we asked to what extent the plasticity observed during learning involves adaptations of key components of the DA system. To this end, we compared mice that had undergone 1 day or 8 days of training on the accelerating rotarod for the expression of DA D1 and D2 receptors as well as of tyrosine hydroxylase (TH) and DA transporter (DAT), the latter two being constituents of the midbrain dopaminergic neurons.

## Materials and methods

### Animals

Eight-teen adult C57Bl6/J male mice (2–4 months old, *N* = 6/group) were used. Animals were purchased from Jackson Laboratory and acclimated to the new environment for at least 2 weeks. The experiment was conducted as a follow up to (Yin et al., 2009), and comprises a new cohort of mice. Animals were housed in standard cages at 21 ± 1°C and 50 ± 5% relative humidity on a 12 h light/dark cycle, with lights on at 7:00 A.M. All procedures were approved by the institutional Animal Care and Use Committee of the National Institute on Alcoholism and Alcohol Abuse, Bethesda, MD in accordance with the NIH Guide for the Care and Use of Laboratory Animals.

### Rotarod training

A computer-interfaced rotarod accelerating from 4–40 rotations per min (rpm) over 300 s was used (ENV-575M, Med-Associates). As previously described, animals were trained with 10 trials per day for either 1 day or 8 days (trained every other day) as described in Yin et al. (2009). Each trial ended when the mouse fell off the rotarod or after 300 s had elapsed, and there was a resting period of approximately 5 min between trials. Forty-eight hours after the last training session mice were sacrificed by decapitation, brains quickly removed, frozen in isopentane at −40°C and stored in −80°C.

### Receptor autoradiography

#### Drugs and reagents

[^3^H]SCH23390 (specific activity 60 Ci/mmol, *K*_D_ = 0.7 nM, *B*_max_ = 347 fmol/mg according to Schulz et al. (1985)), [^3^H]Raclopride (specific activity 80 Ci/mmol, *K*_D_ = 2.08 nM, *B*_max_ = 20.0 fmol/mg according to Hall et al. (1990)) and [^3^H]Mazindol (specific activity 17.8 Ci/mmol, *K*_D_ = 18.2 nM, *B*_max_ = 0.0073 fmol/mg (Javitch et al., 1984)) were from PerkinElmer (Massachusetts, USA), bacitracin, bovine serum albumin, ascorbin acid, nomifensine maleate salt from Sigma-Aldrich (St. Louis, MO, USA), and desipramine hydrochloride, SKF, sulpiride from Tocris Biosciences (Bristol, UK). All other reagents were of analytical grade from regular suppliers.

#### Dopamine receptor and dopamine transporter autoradiography

Mice were sacrificed by decapitation 48 h after training, brains quickly removed, flash-frozen in isopentane at −40°C and stored in −80°C. 12 µm cryostat sections at Bregma levels +1.0 mm, +0.4 mm, −3.5 mm according to Paxinos and Franklin (2004) were thaw mounted onto ice-cold gelatin coated slides and stored at −20°C until use.

Dopamine D1 and D2-like receptor autoradiography in mice were done as recently described in Yin et al. (2009). Sections were brought up to room temperature, incubated for 15 min at room temperature in 50 mM Tris-HCl buffer (pH 7.4) containing either 5 mM MgCl_2_, 1 mM EDTA or 1 mM MgCl_2_, 1 mM CaCl_2_, respectively. Sections were transferred into humidified chambers and 800 µl of reaction mix was applied to each slide, followed by incubation for 2 h at 30°C. The reaction mix for D1 receptor autoradiography contained 10 nM [^3^H]SCH23390 in 50 mM Tris-HCl (pH 7.4), 5 mM MgCl_2_, 1 mM EDTA, 0.1 mM bacitracin and 0.1% bovine serum albumine, and for D2 receptor autoradiography 5 nM [^3^H]raclopride in 50 mM Tris-HCl (pH 7.4), 120 mM NaCl, 5 mM KCl, 1 mM MgCl_2_, 1 mM ascorbin acid, 1 mM CaCl_2_. Nonspecific binding was measured on adjacent sections with addition of either 1 µM SKF or 30 µM sulpiride. The incubation was stopped by washing the sections for three times 2 min in ice cold buffer (50 mM Tris-HCl, pH 7.4), followed by a dip in ice-cold deionized water.

For dopamine transporter autoradiography sections were pre-washed in ice cold washing buffer (50 mM Tris-HCl (pH 7.9), 300 mM NaCl and 5 mM KCl) for 5 min, incubated with reaction mix containing 4 nM [^3^H]Mazindol, 0.3 µM desipramine in 50 mM Tris-HCl (pH 7.9), 300 mM NaCl and 5 mM KCl for 40 min at 4°C. Non-specific binding was determined by adding 100 µM nomifensine to the reaction mix. After incubation sections were washed two times in ice-cold washing buffer (50 mM Tris-HCl (pH 7.9), 300 mM NaCl and 5 mM KCl ) followed by 30 s in ice cold deionized water.

**Figure 1:**
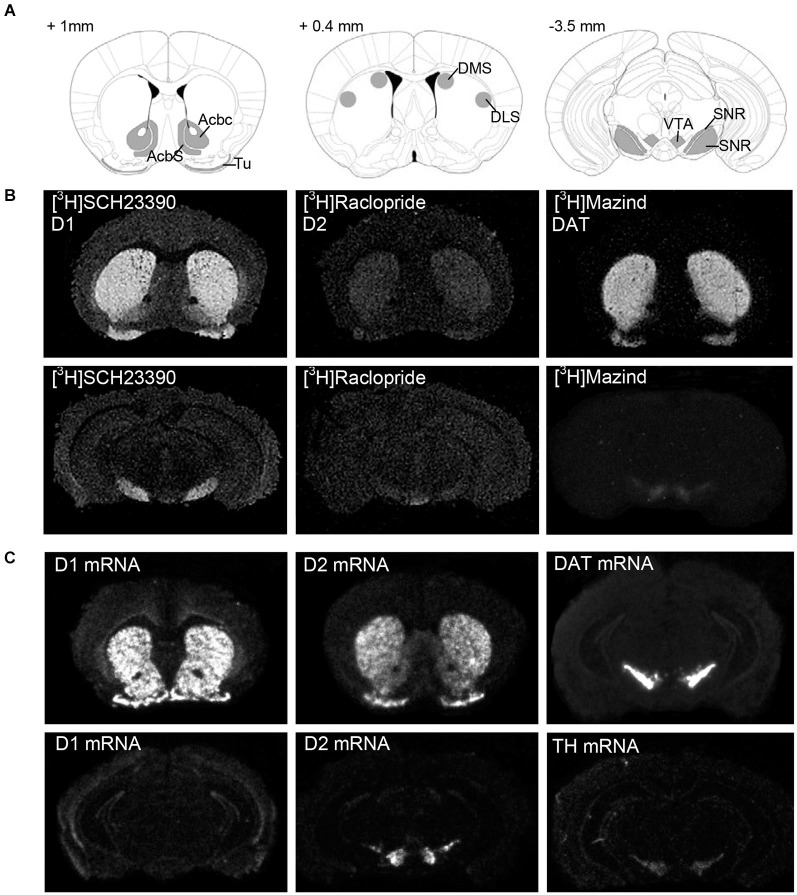
**Representative expression pattern of the dopaminergic neurotransmitter system in a mouse brain. (A)** Schematic representation of the sampled areas for densitometric evaluation of the autoradiography and *in situ* hybridization experiments in a coronal section of a mouse brain. Bregma levels = +1 mm, +0.4 mm, −3.5 mm. AcbC: Accumbens nucleus, core; AcbS: Accumbens nucleus, shell; DLS: Dorsolateral striatum; DMS: Dorsomedial striatum; SNc: Substantia nigra, compacta; SNR: Substantia nigra, reticulata; Tu: Olfactory tubercle; VTA: ventral tegmental area. **(B)** Dark-field microphotographs from [^3^H]SCH23390-, [^3^H]Raclopride and [^3^H]Mazindol binding, and **(C)** *in situ* hybridization signal of D1, D2, DAT and TH mRNA on brain sections of naive C57/Bl6 male mice. For details on treatment, see Section Materials and Methods.

All sections were dried under a stream of cold air and phosphor imaging plates (Storage Phosphor Screen BAS-IP TR2025 E Tritium Screen, GE Healthcare Life Sciences, Pittsburgh, USA) were exposed to sections. Phosphor imager (Fujifilm Bio-Imaging Analyzer Systems, BAS-5000, Fujifilm Corp., Japan) generated digital images (Figure 1) and used for densitometric measurements using MCID Image Analysis Software (Imaging Research Inc., UK). Regions of interest were defined by anatomical landmarks according to the mouse brain atlas (Paxinos and Franklin, 2004) and outlined as shown in Figure 1. Signal density was measured as photostimulable luminescence per mm^2^, compared against standard curves generated using [^3^H]-Microscales (Amersham, GE Healthcare Life Sciences, Pittsburgh, USA) and data (nCi/mg) were converted to fmol receptor per mg protein tissue equivalence. The [3H]-quantitation standard curve was used to interpolate the measured optical densities of the tissue equivalent DA receptors and DA transporter densities from sections (Figure 1) into nCi/mg (B). Binding in femtomoles per milligram (fmol/mg) was calculated based on the specific activity of the radioligand and the saturation binding equation (*B* = *B*_max_*[R]/(Kd + [R]), solving for *B*_max_, *B*_max_ = maximal bound receptor/transporter, Kd = receptor affinity, nM). Data were expressed as fmol/mg protein (mean ± SEM). Signal distribution in mouse brain for all ligands in regions of interest is shown in Figure 1.

#### *In situ* hybridization

The following riboprobes were used for *in situ* hybridization: Drd1a (position 70 bp to 1006 bp on rat cDNA, gene reference sequence: NM_012546.2 with 92% homology to corresponding mouse cDNA sequence), Drd2 (position 1227 bp to 1390 bp on rat cDNA, gene reference sequence: NM_012547.1 with 96% homology to mouse cDNA sequence), DAT (positions 7 bp to 3396 bp on rat cDNA, gene reference sequence: NM_RATDOPER with 91% homology to mouse cDNA sequence), TH (position 1594 bp to 1843 bp on rat cDNA, gene reference sequence: NM_012740 with 95% homology to mouse cDNA sequence). Radioactive labeling of riboprobes using UTP-S^35^ (PerkinElmer, Massachusetts, USA) and *in situ* hybridization procedure has been early described in detail (Hansson et al., 2006, 2008).

Hybridized sections were exposed against FUJI imaging plates (Storage Phosphor Screen BAS-IP SR2025 Screen, GE Healthcare Life Sciences, Pittsburgh, USA) for 5 days and scanned using the Fuji BAS-5000 phosphoimager. On the basis of the known radioactivity in the [^14^C]-Microscale standards (Amersham, GE Healthcare Life Sciences, Pittsburgh, USA), densitometric values were converted to nCi/g. Signal distribution in mouse brain for all probes in regions of interest is shown in Figure 1.

#### Statistics

All data are expressed as means ± SEM. Behavioral data were analyzed repeated measurement ANOVA. Region-wise One-way ANOVAs were used to identify treatment responsive brain regions. Correction of the alpha level was made by Holm’s sequential rejection testing procedure (Holm, 1979) with respect to the number of brain regions analyzed. Raw *p*-values are reported and significance is indicated at levels for α < 0.05, α < 0.01 and α < 0.001.

## Results

**Figure 2:**
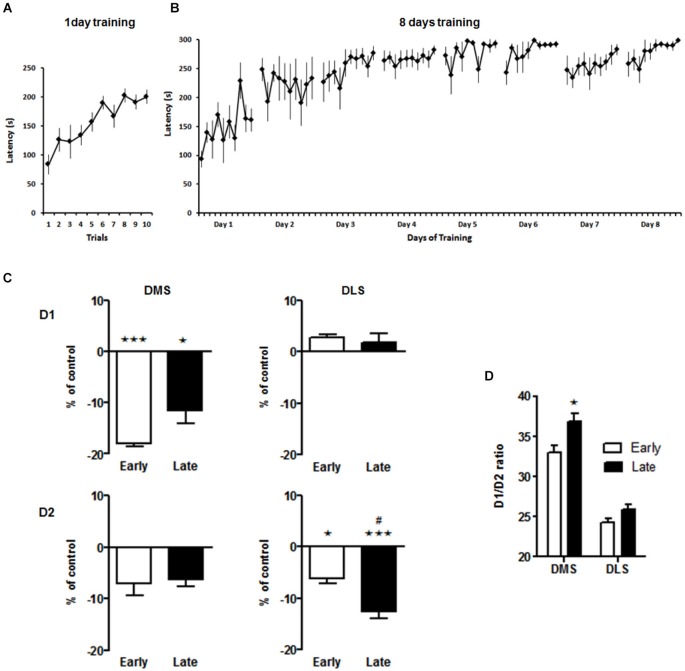
**Spatiotemporal pattern of D1 and D2 binding in the dorsal striatum 1 week after the acquisition or consolidation of skill learning**. Performance of the animals on the accelerating rotarod for the early **(A)** and late **(B)** experimental groups. Latency to fall off the rotarod throughout all training session is shown. **(C)** Ligand binding to D1 and D2 receptors in the dorsomedial (DMS) and dorsolateral (DLS) striatum. Data are presented as percentage (±SEM) of specific binding from the untreated control animals. * *p* < 0.05, *** *p* < 0.001 vs. control group. # *p* < 0.05 early vs. late group. **(D)** Ratio of D1/D2 receptors in DMS and DLS after the early and late training phase. * *p* < 0.05 early vs. late in DMS. For detailed statistics see text.

Three groups of animals (*N* = 6/group) were used. Two groups of mice were trained in the accelerating rotarod (4–40 rpm in 300 s) with 10 trials per day, for either 1 day or 8 days. Training was performed every other day and asymptoted after day 3 of training (Figures 2A,B). Specifically, both groups showed rapid improvement during the 10 trials on the first day (*F*_(5,54)_ = 2.842, *p* < 0.05, *post hoc* first vs. last trial *p* < 0.05), followed by slower improvements across days observed in the group trained for 8 days (*F*_(5.474)_ = 5.710, *p* < 0.01). The third group serving as naïve controls was not exposed to rotarod training, but otherwise handled in parallel with the training groups. Gene expression and ligand binding in relevant brain areas (i.e., Accumbens nucleus core (AcbC), accumbens nucleus shell (AcbS), DLS, DMS, SNC, SNR, olfactory tubercle (Tu), ventral tegmental area (VTA)) were tested in naïve control animals and in groups that had undergone 1 day or 8 days of rotarod training.

Based on our primary hypothesis of specific spatiotemporal neuroplasticiy in the dorsal striatum our initial analysis focussed on effects in the DMS and DLS. We found a down regulation of D1 receptors in the DMS after rotarod training that was most pronounced on day 1, i.e., during the acquisition phase (One-way ANOVA for effect of training: *F*_(2,11)_ = 15.5, *p* < 0.001; *post hoc* tests: early vs. control *p* < 0.001, late vs. control *p* > 0.05, Figure 2C) while D1 binding in the DLS was not affected by the training (One-way ANOVA main effect of training: *F*_(2,12)_ = 1.9, *p* = 0.2). On the other hand, D2-like receptor binding in the DMS was not affected (One-way ANOVA for effect of training: *F*_(2,15)_ = 1.4, *p* = 0.29), but declined significantly with training in the DLS from day 1 to day 8 (One-way ANOVA for effect of training: *F*_(2,13)_ = 14.2, *p* < 0.001; *post hoc* tests: early vs. control *p* < 0.05, late vs. control *p* > 0.001). The net effect of these alterations in DA receptor binding was a significant increase in the D1/D2 ratio in the DMS after extensive training (Two-way ANOVA, main effect brain region *F*_(1,6)_ = 135, *p* < 0.001, main effect training phase *F*_(1,6)_ = 8.3, *p* < 0.03, interaction not significant, Figure 2D).

Compared to the distinct effects of rotarod training on the receptor proteins level, the respective transcripts were barely affected (Table 1). Effects of transcriptional regulation may have taken place at a different time point or been obscured by the intricate circuitry effects. For example, while it is possible to distinguish the postsynaptic and autoreceptor pools of D2 based on the location of their transcripts, the signal of D2 ligand binding in the striatum is a convolute of both.

**Table 1:** **Effects of acquisition and consolidation of motor skill learning on D1, D2, DAT and TH expression levels**.

	Tu	AcbC	AcbS	DMS	DLS	VTA	SNC	SNR
D1 binding fmol/mg
naϊve	3959.8 ± 161.5	1064.5 ± 105.1	1744.3 ± 143.7	4615.3 ± 154.6	4558.3 ± 45.4	301.8 ± 42.7	n.d.	1882.4 ± 84.1
1d	3902.4 ± 75.7	1233.8 ± 68.1	1733.3 ± 180.3	3782.7 ± 21.7 ***	4690.3 ± 29.3	294.0 ± 15.2	n.d.	1702.1 ± 36.1
8d	3172. 8 ± 168.6 **	1766.7 ± 236.2	2820.1 ± 168.0 ***	4080.5 ± 109.2 **	4647.9 ± 82.9	367.8 ± 48.5	n.d.	1574.8 ± 107.2
D1 mRNA nCi/g
naϊve	449.5 ± 19.3	151.0 ± 16.3	281.4 ± 20.5	244.0 ± 3.2	263.5 ± 5.2	7.6 ± 1.0	n.d.	n.d.
1d	464.4 ± 3.3	163.6 ± 10.2	327.9 ± 13.3	239.2 ± 3.2	276.6 ± 7.0	6.3 ± 0.5	n.d.	n.d.
8d	361.2 ± 11.5 ***	156.8 ± 12.9	280.4 ± 12.2	226.2 ± 9.2	261.8 ± 5.4	8.8 ± 1.3	n.d.	n.d.
D2 binding fmol/mg
naϊve	82.6 ± 9.9	54.6 ± 8.0	66.1 ± 11.0	122.0 ± 6.3	205.7 ± 4.2	33.7 ± 3.9	n.d.	33.1 ± 3.1
1d	66.2 ± 0.9	56.9 ± 1.5	82.8 ± 5.8	113.4 ± 2.8	193.1 ± 2.0	26.6 ± 1.4	n.d.	29.6 ± 2.6
8d	88.4 ± 8.1	59.8 ± 3.3	87.8 ± 3.5	114.2 ± 1.5	179.8 ± 2.8 ***	29.3 ± 4.7	n.d.	39.1 ± 1.7
D2 mRNA nCi/g
naϊve	311.6 ± 10.5	108.5 ± 7.3	193.2 ± 6.4	195.1 ± 1.0	257.7 ± 7.6	196.4 ± 5.3	202.3 ± 7.6	n.d.
1d	263.1 ± 9.5 **	108.7 ± 3.1	182.3 ± 4.0	186.8 ± 6.3	239.2 ± 1.9	178.2 ± 8.9	276.1 ± 12.8 ***	n.d.
8d	239.8 ± 10.4 ***	128.8 ± 5.6	216.5 ± 12.4	179.1 ± 6.9	231.3 ± 8.8	185.5 ± 9.9	203.8 ± 9.7	n.d.
DAT binding fmol/mg
naϊve	276.8 ± 20.4	351.10 ± 15.3	190.5 ± 13.1	548.8 ± 29.2	718.2 ± 13.8	249.8 ± 32.2	79.7 ± 9.5	n.d.
1d	171.9 ± 35.1 *	265.0 ± 10.9	110.5 ± 5.3 ***	549.5 ± 17.2	693.2 ± 10.6	216.7 ± 13.7	99.6 ± 6.2	n.d.
8d	339.0 ± 10.7	352.0 ± 31.3	172.0 ± 9.9	502.1 ± 29.8	623.8 ± 30.5	157.0 ± 29.1	94.0 ± 16.2	n.d.
DAT mRNA nCi/g
naϊve	n.d.	n.d.	n.d.	n.d.	n.d.	2215.5 ± 31.5	2260.2 ± 18.5	n.d.
1d	n.d.	n.d.	n.d.	n.d.	n.d.	2159.2 ± 44.7	2183.6 ± 49.4	n.d.
8d	n.d.	n.d.	n.d.	n.d.	n.d.	2078.0 ± 154.0	1688.9 ± 304.2 *	n.d.
TH mRNA nCi/g
naϊve	n.d.	n.d.	n.d.	n.d.	n.d.	52.7 ± 1.7	40.7 ± 1.7	n.d.
1d	n.d.	n.d.	n.d.	n.d.	n.d.	50.8 ± 5.3	40.9 ± 5.0	n.d.
8d	n.d.	n.d.	n.d.	n.d.	n.d.	53.5 ± 2.6	48.8 ± 4.5	n.d.

Data are expressed as mean ± SEM. Statistical analysis was performed by region-wise 1-way ANOVAs followed by multiple testing correction using Holm’s corrected Bonferoni’s post hoc test. Corrected p-values: *p < 0.05, **p < 0.01, ***p < 0.001 vs. naïve control group; n.d. not detected. AcbC: Accumbens nucleus, core; AcbS: Accumbens nucleus, shell; DLS: Dorsolateral striatum; DMS: Dorsomedial striatum; SNC: Substantia nigra, compacta; SNR: Substantia nigra, reticulata; Tu: Olfactory tubercle; VTA: ventral tegmental area.

Considering the overall picture of DA systems adaptations during task acquisition and consolidation across a number of relevant brain regions, we found few significant alterations in DA receptor expression and binding outside of the dorsal striatum (Table 1). Notably, D1 receptor binding was significantly increased after 8 days of rotarod training in the nucleus accumbens shell region and decreased in the Tu. D2 receptor mRNA was significantly decreased in the Tu and increased in the early phase of training in the dopaminergic cells of the SNC, the latter potentially reflecting an early effect on the expression of D2 autoreceptors, which at the level of ligand binding can not be distinguished in the striatal projection area from D2 binding to dopaminoceptive medium spiny neurons. Dopamine transporter binding in terminal regions of the DA neurons was significantly decreased by 1 day of training in the Tu and the nucleus accumbens shell. After longer training there was a significant downregulation of DAT mRNA expression in the DA neurons of the SNC. Thyroxin hydroxylase expression was not significantly affected by the training.

## Discussion

By investigating striatal D1-like and D2-like receptor binding after initial and prolonged training of mice in the accelerated rotarod task, we provide further evidence for the differential involvement and plasticity of DMS and DLS receptor populations during acquisition and consolidation of this skill. We observed that the distinct spatiotemporal pattern of neural activity in the dorsal striatum during learning of a simple motor skill as described in our previous study (Yin et al., 2009) is generally mimicked by adaptations of dopamine D1 and D2 receptors in these neurons. Specifically, D1 neurons in the DMS, which are preferentially active in the early phase of training, show an early reduction in D1 binding, while D2 neurons in the DLS, known to be increasingly engaged with skill consolidation, show a progressive reduction in D2 binding.

Although our pharmacological tools targeted D1-like and D2-like binding, the measurements from the dorsal striatum are representative of the D1 and D2 receptor containing neuronal populations, because these subtypes comprise the vast majority of dopamine receptors within this region (Gerfen et al., 1990; Diaz et al., 2000; Gangarossa et al., 2013). These studies found only sparsely distributed D3-receptors in the dorsal striatum. However, given that this D3-receptor population is regulated following behavioral or pharmacological manipulations (Guillin et al., 2001; Jeanblanc et al., 2006), we cannot exclude that D3 may account for some of the changes in D2-like binding. Furthermore, it is important to note that the measurements were taken 2 days after the last respective training session. This time point was chosen to avoid acute effects on receptor trafficking during task performance. On the other hand, while the receptor proteins show robust changes at this time point, transcript levels were largely unaltered. This dissociation in protein and mRNA changes could reflect different kinetics, i.e., mRNAs typically have a much faster turnover and regulatory response compared to proteins, or may indicate the involvement of mostly post-transcriptional regulatory mechanisms under the present experimental conditions. In any case, a short, about 2 h intense training period leaves a profound mark on the number of D1 receptors presented by DMS neurons. This effect seems to diminish with task consolidation. On the other hand, D2 neurons in the DLS show a progressive loss of their D2 receptors with prolonged training. Together it appears that the specific roles of D1-DMS and D2-DLS neuronal populations in learning of the skill are associated with decreased ability of these neurons to respond to dopaminergic input.

Outside of the dorsal striatum we found only few notable DA systems adaptation. These include opposing effects on D1 binding between nucleus accumbens shell and Tu after extended training as well as reduction in DAT binding sites in both the nucleus accumbens shell and the Tu after 1 day of training. The Tu is a major site of dopaminergic innervation, considered as a part of the ventral striatum and strongly involved in associative learning and response strategy selection (Ikemoto, 2007). The functional implications of these various phenomena for the rotarod task remain obscure. However, learning a new skill can be considered as stressful, while on the other hand, exercise by itself is rewarding. Both processes are expected to result in adaptive responses in the reward system.

In our previous experiments we found different types of neuroplasticity during early and late training phases. In the DMS potentiation of synaptic strength was observed only in the early training phase, with extended training resulting in a return of synaptic strength back to naïve levels. In contrast synaptic strength developed gradually with extended training in the DLS resulting in long lasting potentiation of glutamatergic transmission (Yin et al., 2009).

Dopamine facilitates synaptic plasticity in both D1 and D2 receptor expressing striatal neurons (Shen et al., 2008). However, the issue is complex and it is unclear to what extent the dynamics of receptor change are related to an involvement of the cells expressing them in performing the skill. For example, D2 receptors are supposedly negative modulators of striatopallidal cells, and therefore downregulation of D2 surface receptors in DLS with training may reflect an increase in engagement of these cells. Alternatively or additionally, the decrease in D2 surface receptors may be a consequence of these cells becoming more engaged (hence the downregulations of this negatively modulating receptor).

Dissociation between striatal subregions and neuronal subtypes in the regulation of motor behavior has been demonstrated under a variety of experimental conditions including pharmacological or genetic lesions, systemic or site-specific pharmacological manipulations and pathway-specific optogenetic stimulation (Durieux et al., 2009, 2012; Yin et al., 2009; Bateup et al., 2010; Kravitz et al., 2010; Lobo et al., 2010). The observed behaviors differ between experimental approaches and may seem at odds with our data in an intact animal model. However, these studies converge in supporting a role of the DMS in promoting fast responses and of the DLS in long-term plasticity.

Under normal conditions, the output of striatonigral D1 and striatopalidal D2 neurons is well balanced, while severe distortion of this balance or unilateral neuroplasticity is associated with motor and behavioral symptoms (Albin et al., 1989; Sommer et al., 1993, 1996; Shen et al., 2008; Gerfen and Surmeier, 2011; Cui et al., 2013). Here, we observed a significant increase in the D1/D2 ratio in the DMS after long-term training. It remains to be determined if tilting the balance towards D1 after a period of overtraining is an adaptation to the increased motor activity or could prepare the brain for learning new motor sequences. Our experiment also underlines the importance of studying intact animals for elucidating learning and performance related plasticity.

Importantly, different roles of DMS and DLS are observed not only in motor but also procedural learning, where both regions are mediating different action strategies with the DMS being necessary for goal-directed behavior and the DLS mediating habitual responses (Gremel and Costa, 2013). Furthermore, extended alcohol self-administration in rats produces habit-like responding, while response control shifts from the DMS to the DLS across the course of training (Corbit et al., 2012). Although direct evidence from humans is lacking so far, it is likely that the transfer of control from medial to lateral striatal compartments is important for behavioral pathologies in humans. Among alcohol drinkers those with moderate consumption show activation of medial striatal regions (including the caudate nucleus) in a functional magnetic resonance imaging (fMRI) paradigm upon respective cue presentation, which probably reflects an outcome-oriented response, while heavy consumers or addicts with strongly automated behaviors lack such activation but engage more dorsolateral parts (putamen) of the striatum (Vollstädt-Klein et al., 2010).

In conclusion, the present results are important for understanding the neurochemical balance in the striatum during learning, and potentially may help to device pharmacological interventions that can help to break bad habits.

## Conflict of interest statement

The authors declare that the research was conducted in the absence of any commercial or financial relationships that could be construed as a potential conflict of interest.
